# Strand-Specific RNA-Seq Provides Greater Resolution of Transcriptome Profiling

**DOI:** 10.2174/1389202911314030003

**Published:** 2013-05

**Authors:** James Dominic Mills, Yoshihiro Kawahara, Michael Janitz

**Affiliations:** 1 School of Biotechnology and Biomolecular Sciences, University of New South Wales, Sydney, NSW 2052, Australia; 2 National Institute of Agrobiological Sciences, Agrogenomics Research Center, Bioinformatics Research Unit, 2-1-2 Kannondai, Tsukuba, Ibaraki 305-8602, Japan

**Keywords:** Antisense RNA, Next-generation sequencing, Non-coding, RNA, Transcriptome, Pervasive transcription.

## Abstract

RNA-Seq is a recently developed sequencing technology, that through the analysis of cDNA allows for unique insights into the transcriptome of a cell. The data generated by RNA-Seq provides information on gene expression, alternative splicing events and the presence of non-coding RNAs. It has been realised non-coding RNAs are more then just artefacts of erroneous transcription and play vital regulatory roles at the genomic, transcriptional and translational level. Transcription of the DNA sense strand produces antisense transcripts. This is known as antisense transcription and often results in the production of non-coding RNAs that are complementary to their associated sense transcripts. Antisense tran-scription has been identified in bacteria, fungi, protozoa, plants, invertebrates and mammals. It seems that antisense tran-scriptional ‘hot spots’ are located around nucleosome-free regions such as those associated with promoters, indicating that it is likely that antisense transcripts carry out important regulatory functions. This underlines the importance of identifying the presence and understanding the function of these antisense non-coding RNAs. The information concerning strand ori-gin is often lost during conventional RNA-Seq; capturing this information would substantially increase the worth of any RNA-Seq experiment. By manipulating the input cDNA during the template preparation stage it is possible to retain this vital information. This forms the basis of strand-specific RNA-Seq. With an ability to unlock immense portions of new in-formation surrounding the transcriptome, this cutting edge technology may hold the key to developing a greater under-standing of the transcriptome.

## 1. INTRODUCTION

As sequencing techniques become more sophisticated and our understanding of molecular biology increases, it has become apparent that the pathway from gene to protein is an intricate and multifaceted process. Organism complexity highlights this; there is no correlation between genome size or number of genes and the complexity of an organism [1]. Intuitively, it would be thought that increased organism complexity would require a larger number of protein coding genes; this is not the case. A human genome contains approximately 30,000 protein coding genes, the fruit fly *Drosophila melanogaster *has approximately half this amount, while the salamander has twenty times this number of genes [2-4]. One trend that does exist amongst higher eukaryotes and indeed humans is an increase in alternative splicing events and the addition of a variety of non-coding RNAs (ncRNAs) [5]. Up 98% of the transcriptional output of the genome is made up of ncRNAs. It was initially thought that this extra transcriptional output was the result of misdirected transcription, however it seems unlikely that such an energy consuming and inefficient system would become prevalent. It is now thought that these non-coding RNAs play major regulatory roles and are involved in chromatin remodeling, RNA-DNA RNA-RNA, and RNA-protein interactions as well as other unknown forms of regulation [5-8]. It is thought that this complex network of regulatory elements plays an important role in the development of organism complexity. This highlights the importance of research in the field of transcriptomics and more specifically ncRNAs.

One component of the transcriptome of particular interest is transcription from the DNA sense (or plus) strand. Transcription from this strand produces transcripts known as antisense transcripts or antisense RNAs (asRNAs) [9]. Currently there is a very limited body of research in this area. Antisense transcription can produce both protein and non-coding transcripts, with the latter being the most common product of antisense transcription. As antisense transcription is a pervasive feature of the mammalian transcriptome, it is likely that these transcripts play important regulatory roles [9, 10]. Furthermore the act of antisense transcription itself can have a regulatory function. Importantly there are a number of antisense transcripts transcribed from genes that are related to various human disorders [10]. It has also been suggested that asRNAs may play an important role during development and changing environmental conditions by altering expression patterns [11-13]. A full elucidation of the antisense transcriptome will open up new levels of understanding that may help develop insight into the complex working of the transcriptome.

RNA-Seq is a next-generation sequencing technique that allows for an in depth look into the transcriptome [14]. It is perhaps the most exciting next-generation sequencing application. It is has many advantages over other methods of transcriptome analysis such as microarrays, and is adept at identifying alternative splicing events and ncRNAs [14, 15]. While the use of RNA-seq is becoming more common throughout molecular biology, one significant short-coming of the standard RNA-seq protocol is that it loses the strand of origin information for each transcript. This is of particular concern due to the possible regulatory role carried out by antisense transcripts. It is possible to retain this information pertaining to strand origin by modifying the standard RNA-Seq protocol; this is known as strand-specific RNA-Seq [16]. Perhaps, due to the increase in the time and knowledge required or lack of awareness, this method is severely underutilized in research. This could be leading to vital elements of the transcriptome being overlooked. Through the use of strand-specific RNA-Seq a more complete understanding of the transcriptome could be achieved, this has the potential to identify new levels of regulation of gene expression.

## 2. ANTISENSE TRANSCRIPTS

### 2.1. Antisense Transcription

DNA exists as a double stranded molecule; one strand is known as the sense (or plus) strand the other is known as the antisense (or minus) strand. The antisense strand contains all of the pertinent information for the formation of proteins; it was originally thought that this strand was the only strand that underwent transcription. Transcription from the sense strand is less common and it will produce what is knows as antisense transcripts or asRNAs. It was previously thought that this form of transcription was an aberration of the norm, however antisense transcription has been identified at higher then expected levels in prokaryotes and eukaryotes; including humans and other mammals [9, 17-21]. Antisense transcription can produce antisense transcripts that are polyadenylated and undergo the addition of a 5’ cap, which then proceed to the ribosomes for translation. However, the most common form of antisense transcription in the mammalian genome is the production of non-coding antisense transcripts that have a protein coding sense partner [9]. Furthermore, antisense transcripts have been documented that partner with active promoter sites or those that are in close proximity of transcription start sites [17, 22, 23]. While antisense transcripts occur at lower abundances than their sense transcripts, all evidence points to non-coding antisense transcripts playing a pivotal role in regulation of the transcriptome [19]. 

### 2.2. Antisense Transcripts As Regulators of the Transcriptome

There exist a variety of pathways in which antisense transcripts can act as regulatory elements. It is possible to divide these pathways into three broad categories; transcription modulation, hybridization of sense-antisense RNA partners and chromatin modification. As the field of non-coding RNA is still relatively young and the field of asRNA comparatively unexplored, it is likely that more mechanisms of regulation by these elements will be elucidated in the future.

#### 2.2.1. Transcription Modulation

The act of antisense transcription, rather than asRNA molecule itself can modulate gene expression levels. During transcription RNA polymerase binds to the promoter region of the gene and proceeds along the strand. If transcription occurs on the DNA sense strand and antisense strand simultaneously it can result in the RNA polymerases colliding. This is known as the transcriptional collision model [10]. While it is not exactly known how colliding RNA polymerases interact, it would most likely result in either complete termination of transcription or the termination of transcription on one strand [24]. Transcriptional collision has been observed in *Saccharomyces cerevisiae *and bioinformatic studies have suggested that it does occur in mice and humans [25, 26]. The study of mice and humans indicates that a converse relationship between antisense and sense transcripts exists. Furthermore, this relationship was stronger when the antisense-sense pair shared a longer section of DNA [25]. As expected the longer the common region between an antisense-sense pair, the more likely it would be for transcriptional collision to occur.

#### 2.2.2. Hybridization of Sense-Antisense RNA Partners

Antisense transcripts are complementary to their sense partners; this means that there is always a chance of hybridization, and the formation of a RNA duplex. Duplex formation can exert regulatory functions in diverse ways. Factors that impact on the regulatory implications of the RNA duplex include length of hybridization, location along the RNA transcript of hybridization and whether the duplex forms in the nucleus or the cytoplasm. The formation of RNA duplexes is probably the main way in which antisense transcripts exert regulatory functions.

Antisense transcripts have the potential to mask many regulatory components of sense RNA transcripts. Alternative splicing is a process by which exons and introns of primary transcripts can be included, excluded or skipped to form unique mRNAs. Splicing is controlled by the presence of exonic splicing enhancers/silencers and intronic enhancer/silencers, the ratios of these elements impact on the splicing pattern [27]. These elements contain motifs that will recruit splicing machinery to the site. If sections of the transcript containing these elements are masked, by hybridization with an antisense transcript, then the splicing patterns of the sense transcript will be changed [10]. For example, the thyroid hormone receptor-α gene (*THRA*) has splicing patterns that can be altered by the presence of its antisense transcript partner [28]. It can be postulated that a similar mechanism could also block the binding of miRNA to mRNA and hence alter the regulation of the transcripts [10]. The number and pattern of splicing elements and miRNA binding sites that are masked would depend on the length and positioning of the asRNA. Changing the number of these sites available could dramatically alter the functionality of the RNA and its protein counterpart. Furthermore, antisense transcription has been highlighted at elevated levels near promoters and transcription start sites hence a similar mechanism could also alter the function of these elements.

RNA duplex formation in the cytoplasm may alter the ability of a transcript to be translated. It is possible that the duplex formation blocks the ability of the transcript to associate with the ribosome hence altering the efficiency of the translation machinery. In the case of the gene spleen focus forming virus proviral integration oncogene spi1 (*SPI1* or *PU.1*) its non-coding antisense transcript partner stalls translation between the initiation and elongation steps [29]. Further, cytoplasmic RNA duplex formation can also alter the stability of the mRNA. The gene β-site amyloid precursor protein (APP)-cleaving enzyme (*BACE1*) has been associated with Alzheimer’s disease (AD) [30]. It plays a pivotal role in the cleavage of the amyloid precursor protein (*APP*), leading to a build up amyloid-β peptides. It has been shown that the antisense transcript of *BACE1* is up-regulated in AD brains [31]. It has been reported that the *BACE1 *antisense transcript and *BACE1 *will form a duplex and this increases the stability of the transcript through the formation of secondary and tertiary structures. Formation of these secondary and tertiary structures protects the transcript from degradation while still allowing it to function as a cleaving enzyme. Furthermore, as amyloid-β peptides levels are increased the levels of *BACE1* antisense transcripts are also elevated, creating a positive feedback loop [31].

#### 2.2.3. Chromatin Modification

Genomic DNA in eukaryotic cells must be assembled appropriately to fit within the nucleus of the cell. Generally, genomic DNA in its packaged state exists as 146bp segments tightly encompassing a histone protein octamer and these units are known as nucleosomes [32]. Each nucleosome is connected by a short DNA fragment, this forms a structure known as chromatin, the chromatin interacts with itself and condenses even further to form chromosomes [33, 34]. Evidence has emerged that the structure of the nucleosomes can vary, and this structure impacts on transcription, replication and DNA repair [34, 35]. 

It is thought that long non-coding RNAs are involved in the regulation of chromatin, possessing the ability to remodel the nucleosome complex [24, 36]. Histone modifying enzymes lack the specific DNA binding domains that are seen in other transcription factors [37]. It has been suggested that long ncRNAs, such as those produced by antisense transcription, may interact with histone modifying enzymes via the formation of specific RNA secondary structures [36]. In this case long non-coding antisense transcripts may act a recruitment vessels for various histone remodeling enzymes. This complex will then alter chromatin structure by adding, removing or replacing various chromatin modifications [24, 36].

Perhaps the best example of an antisense transcript being involved in chromatin remodeling is X-inactivation [38]. X-inactivation is achieved in mammals through regulation of the chromosome by the gene X-inactive specific transcript (*XIST*) [39]. The highly expressed *XIST *coats one of the X-chromosomes, the coated X will go on to become the inactive X [40]. The asRNA partner, of *XIST* known as *TSIX, *is a 40 kb RNA that is located 15 kb downstream of *XIST* [38]. *TSIX *is suspected to play a role in X-inactivation, by keeping *XIST *from coating both X-chromosomes. It is suspected that *TSIX *silences *XIST *through the modification of the chromatin structure by recruiting protein complexes that are involved in heterochromatinization [41]. If *XIST *is repressed by *TSIX *it follows that the X-chromosome will remain active. Strand specific FISH probes have demonstrated that *TSIX *expression is associated with the active X, while no *TSIX *is found in cells that have entered the X-inactivation pathway [38].

## 3. RNA-SEQ ANALYSIS

RNA-Seq is a recently developed next-generation sequencing technology, that through the analysis of cDNA allows for unique insights into the transcriptome of a cell. The data generated by RNA-Seq provides information on gene expression, alternative splicing events, locations of transcription factor binding sites and the presence of non-coding RNAs. It has been realised non-coding RNAs are more than just artefacts of erroneous transcription and play vital regulatory roles at the genomic, transcriptional and translational level. It is thought that these additional levels of regulation are the greatest contributors to the complexity seen in higher eukaryotic organisms such as humans [5, 42]. This makes the identification, annotation and cataloguing of mRNA transcripts, non-coding RNAs, microRNAs (miRNAs) and their associated binding sites one of the major aims of the field of transcriptomics [43]. RNA-Seq plays a key role in completing this goal. A variety of next-generation sequencing high throughput platforms can be used for RNA-Seq including systems from Roche, Illumina and Applied Biosystems [44].

The data generated by RNA-Seq can then be analyzed using free, open source bioinformatics software tools such as TopHat and Cufflinks [45]. TopHat, which is a popularly used splice junction-aware mapping tool, aligns the RNA-Seq to a genome of choice, and Cufflinks then assembles transcripts and estimates expression levels using alignments and splice junction information. The bioinformatics analysis can be extended through the use of Cuffdiff. Through the use of statistical models Cuffdiff highlights the genes that are differentially expressed between two or more samples [45]. These tools can be applicable to both standard and strand-specific RNA-Seq data by specifying appropriate options. For the most up to date RNA-Seq bioinformatic analysis workflow description the reader is directed to the protocol paper by Trapnell *et al*., 2012 [45]. Open, web-based platforms such as Galaxy (http://galaxyproject.org/) have been developed to make these programs and various other more complex tools user friendly and accessible to all wet-lab research scientists even those with little or no bioinformatics training [46-48].

RNA-Seq has several advantages over other gene expression quantification platforms such as exon arrays and reverse transcription quantitative real-time PCR. RNA-Seq is a high throughput method that requires low levels of initial input RNA, it has a high resolution in terms of determination of transcripts structure and their quantification, low levels of background noise and produces fewer false positive results [43]. Furthermore, RNA-Seq is able to detect novel, unannotated transcripts, can reveal the exact location of transcript boundaries and determine single nucleotide polymorphisms in transcribed regions [49]. There is also a growing body of evidence demonstrating high accuracy and reproducibility of RNA-Seq [50]. The major challenges faced by RNA-Seq concern storage, retrieval and processing of large amounts of data. However, with the advent of cloud computing these issues are becoming increasingly inconsequential [51].

## 4. STANDARD RNA-SEQ PROTOCOL

Despite differences in sequencing reaction chemistries and base calling all of the RNA-Seq platforms share commonalities in terms of major steps in template preparation [43]. The construction of a cDNA library is the first step in any RNA-Seq workflow. To construct a cDNA library an appropriate RNA fraction must be selected from the total RNA preparation. The total RNA of an organism contains approximately 90% ribosomal (rRNA) [52], for downstream RNA-Seq analysis it is therefore important to remove this potential contaminant from the sample. This can be done in two ways; selection of mRNA transcripts by using oligo(dT) primers or through rRNA depletion [52]. It is important to realize that oligo(dT) primers only select for polyadenylated mRNA. As the transcriptome is made up of numerous RNAs species that are non-polyadenylated; including preprocessed RNA, tRNA, numerous regulatory RNA molecules, and other RNA molecules of unknown function, this method could lead to development of rather simplified picture of the transcriptome [6, 14, 52]. The second method, rRNA depletion can remove up to 99.9% of all large rRNA molecules, while leaving all mRNA and non-polyadenylated transcripts intact. Illumina (San Diego, California) employs a Ribo-Zero kit for the removal of rRNA during RNA sample preparation (http://www.illumina.com/products/truseq_rna_sample_prep_kit_v2.ilmn). This method makes use of a bead capture procedure, where the beads will selectively bind rRNA molecules, the RNA bound beads are then removed using a magnet. This leaves the diverse RNA species representing an intact transcriptome.

After selection of the appropriate RNA fraction is completed, the molecules must be fragmented into smaller pieces, to a size between 200-500bp depending on the sequencing platform being used [43]. This fragmentation can be achieved in two ways; fragmentation of double-stranded (ds) cDNA or the fragmentation of RNA. Both methods result in the same end product of a double stranded cDNA library in which each fragment has an adapter attached [14]. 

For the direct fragmenting of double stranded (ds) cDNA, first the RNA must be reverse transcribed. This can be done using either random hexamers or oligo(dT) primers. Again oligo(dT) primers will fail to pick up any RNAs that are not polyadenylated. The ds cDNA is then fragmented and primer adapters are ligated [14]. Alternatively, direct fragmenting of RNA allows for a much more in-depth transcriptome analysis. First the RNA is fragmented, this is followed by two rounds of cDNA synthesis (first strand and second strand) using random hexamers, and adapters are then ligated to the ds cDNA [14]. Once the ds cDNA libraries are constructed they are sequenced producing short reads. The sequencing can be either single-end or paired-end. The Illumina (San Diego, California) RNA-Seq protocol utilizes direct fragmentation of RNA followed by reverse transcription by random hexamers (http://www.illumina.com/products/tru seq_rna_sample_prep_kit_v2.ilmn).

## 5. STRAND-SPECIFIC RNA-SEQ

The standard method of RNA-Seq library generation fails to preserve the information pertaining to which DNA strand was the original template during transcription and subsequent synthesis of the mRNA transcript. Since antisense transcripts are likely to have regulatory roles that are distinctly different from their protein coding complement, this loss of strand information results in an incomplete understanding of the transcriptome [16, 17, 53]. To this end, it is possible to retain the strand origin of various transcripts using a method known as strand-specific RNA-Seq. While more challenging technically and more time consuming then standard RNA-Seq [16], the extra information gathered from a strand-specific RNA-Seq experiment cannot be overlooked. Strand-specific RNA-Seq allows for sense and antisense transcript structures to be predicted, overlapping regions of transcription can be identified exactly and expression levels of sense and antisense genes can be more accurately estimated.

**Fig. (1):**
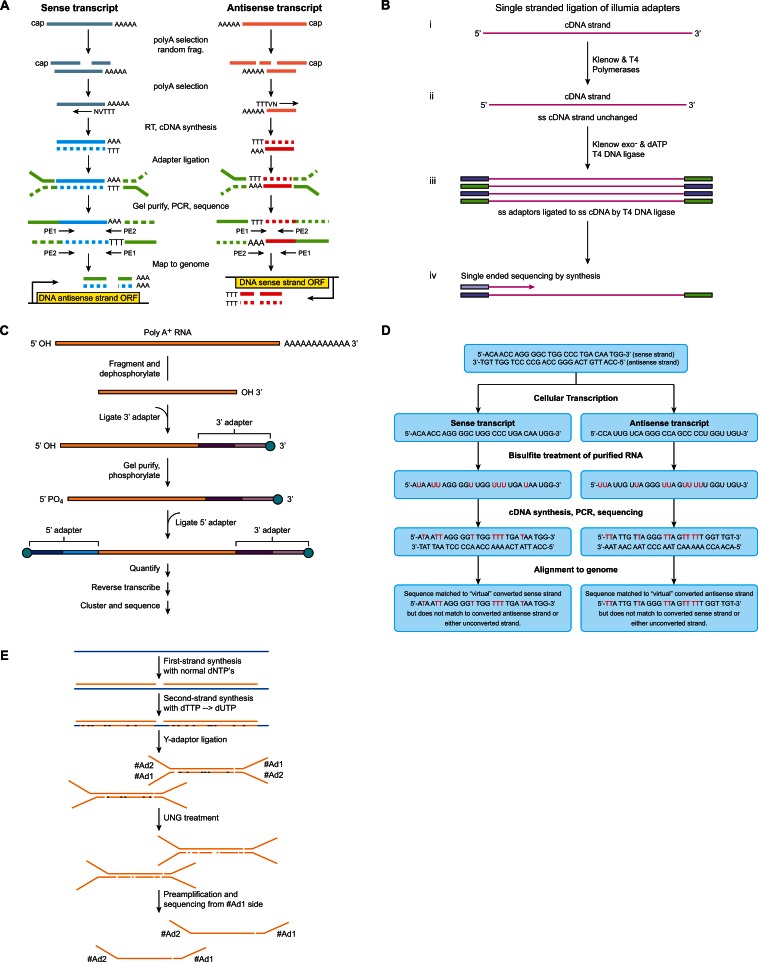
Different methods of template preparation for strand-specific RNA-Seq. A. Strand-specific 3’-end RNA-Seq. Reads that align with a stretch of adenines at the end of the transcript are sense transcripts originating from the DNA antisense strand. Reads that align with a stretch of thymines at the front of the transcript are antisense transcripts originating from the DNA sense strand [54]. B. Single-stranded adapter ligation. Adapters are ligated directly to ss cDNA using T4 DNA ligase. As there is only one strand, stranded information is retained [56]. C. FRT-Seq. Poly(A) RNA is selected and fragmented. Adapters are ligated to the 3’ and 5’ end. The adapters consist of two regions; for the 3’ adapter the light purple region hybridizes to the flowcell surface and the sequencing primers anneal to the dark purple region. Similarly for the 5’ adapter the dark blue region hybridizes to the flowcell and the sequencing primers anneal to the light blue region. The fragments undergo reverse transcription on the flowcell surface then proceed to sequencing [59]. D. Bisulfite Treatment. By applying bisulfite mix to the RNA strand, all cytidine residues are converted to uridine. Through subsequent alignment with converted sense and antisense strands, the strand of origin can be identified. Reads from sense transcripts will align with the converted DNA sense (plus) strand, but not with the converted DNA antisense (minus) strand or either of the unconverted strands. Reads from antisense transcripts will align with the converted DNA antisense (minus) strand, but not converted DNA sense (plus) strand or either of the unconverted strands [19]. E. dUTP second strand. During second strand synthesis dUTP are added to the mix rather than dTTPs. These residues are removed by the addition of UNG (also known as UDG), destroying the strand. Only one strand proceeds to sequencing [63].

Methods for the construction of strand-specific RNA-Seq libraries can be split into two categories (i) the use of known orientation strand-specific adapters, and (ii) the chemical modification of strands [16]. The main methods discussed in this paper are summarized and compared in Table **1** and Figure **1**.

### 5.1. Adapter Methods for Stand-Specific RNA-Seq

Adapter methods for strand-specific RNA-Seq are conceptually difficult and require more consideration and planning when compared to standard RNA-Seq. There has been an array of different protocols outlined. These methods utilize the known sequences at the 5’ or 3’ end and the relative orientations of the RNA transcript to derive strand information.

In the strand-specific 3’-end RNA-Seq method anchored oligo(dT) primers are first used to select for mRNA, which results in production of double-stranded cDNA molecules [52]. Adapters for paired end sequencing are then ligated to each end of the cDNA molecule. Subsequently, the fragments are sequenced generating pair-end reads that are aligned to a reference genome. Any aligned read that contains a stretch of adenines at the end of the transcript must be a transcript that originated from the DNA antisense strand, while any reads that align with a stretch of thymines at the front must be a transcript from the DNA sense strand (Fig. **1A**) [54]. This protocol while reasonable for identifying those antisense transcripts that are capped and polyadenylated, will miss out on the diverse repertoire of non-processed RNAs. Due to the use of oligo(dT) primers for the selection of RNA. This constitutes a significant shortcoming of this protocol as the main driving force behind strand-specific RNA-Seq is to identify non-coding RNAs, which may or may not be capped and polyadenylated [55]. It is also foreseeable that alignment and identification of strand origin may be difficult when sequencing organisms with A-T rich transcriptomes.

Another strand-specific RNA-Seq protocol makes use of single-stranded (ss) cDNA and Illumina adapters (Fig. **1B**) [56]. The standard RNA-Seq protocol requires the input of ds cDNA and through the generation of this ds cDNA library the strand-specific information is lost. Application of T4 DNA ligase allows for linking of 3’ and 5’ adapters to ssDNA [57]. These single-stranded constructs can then undergo sequencing. As the second strand is never synthesized and does not proceed to sequencing, strand information is retained. This can also be seen as a simplification of the standard RNA-Seq method as there is no need for second strand cDNA synthesis. An analysis of the single-stranded adapter ligation RNA-Seq protocol demonstrated that the results produced are comparable to those produced by the standard RNA-Seq protocol [56]. 

Flowcell reverse transcription sequencing (FRT-Seq), is another category of RNA-Seq that maintains strand information. Of all the methods discussed it diverges the greatest amount from the standard RNA-Seq protocol, but it can be loosely classified as an adapter method (Fig. **1C**). [58, 59]. FRT-Seq involves the ligation of specially designed adapters to either end of fragmented and purified polyadenylated mRNA. Each adapter consists of two regions; a region to which the sequencing primers anneal and a region that is complementary to the oligonucleotides present on the flowcell [59]. This complementary region allows the mRNA fragment to hybridize to the flowcell. The mRNA fragments are then reverse transcribed on the flowcell surface. Once this has been completed cluster amplification and sequencing proceeds as normal. This method avoids library amplification and all of the possible biases, including unequal amplification efficiencies of different RNA-species and the introduction of amplification artifacts. For this protocol to work properly it requires a much larger initial RNA input [59].

There are other adapter methods for strand-specific RNA-Seq. These include direct strand-specific sequencing (DSSS) developed by Vivancos, *et al.* 2010 [60], which is based on the Illumina small RNA sample preparation protocol (http://www.illumina.com/products/truseq_small_rna_sample_prep_kit.ilmn), and the Life Technologies (California, USA) SOLiD^®^ Total RNA-Seq Kit that preserves strand specificity through the addition of adapters in a directional manner (http://products.invitrogen.com/ivgn/product/ 44453 74).

### 5.2. Strand-Specific RNA-Seq by Chemical Modification

Another method of carrying out strand-specific RNA-Seq involves the marking the RNA strand through chemical modification, either on the RNA itself or during second strand cDNA synthesis [16]. These methods are the most commonly used methods for strand-specific RNA-Seq and involve only a slight deviation from the standard RNA-Seq protocol.

The first method of chemical modification involves marking the original RNA template through the use of bisulfite treatment (Fig. **1D**). Through the addition of bisulfite mix, all cytidine residues of the RNA strand are changed to uridine residues [19, 61]. This creates a purified RNA strand that contains an artificially high number of uridine residues. After cDNA synthesis and sequencing the resultant reads will contain a high proportion of deoxythymidine residues. These reads are then aligned to a converted plus and minus strand reference genome, where all cytosine residues have been converted to thymine residues. In this case reads from sense transcripts will align with the converted plus strand, but not with the converted minus strand or either of the unconverted strands. Likewise, reads from antisense transcripts will align with the converted minus strand, but not converted plus strand or either of the unconverted strands [19]. In this way the transcripts strand of origin is retained. 

The second method of chemical modification, known as dUTP second strand, involves a modified second-strand synthesis step. During this step dUTPs are incorporated into reverse transcription reaction, this results in ds cDNA where the original strand has deoxythymidine residues while the complementary strand contains deoxyuridine residues. Through the use of Uracil-DNA-Glycosylase (UDG) these residues are degraded, which leaves the first strand intact (Fig. **1E**) [62-66].

When sequencing proceeds the polarity information from the original RNA molecules is maintained. A recent protocol by Zhang *et al.*, 2012 [66] further optimizes this technique by using a Ribo-zero kit for RNA selection. Illumina’s TruSeq^®^ Stranded Total RNA Sample Prep Kit (http://www.illu mina.com/ products/truseq_stranded_total_rna_sample_prep _kit.ilmn) uses a similar method to maintain strand information. For the Illumina protocol second-strand synthesis also involves the incorporation of dUTPs, however instead of using UDG to degrade the residues a special polymerase is used during amplification that will not incorporate these nucleotides. In a comprehensive comparison of various strand-specific RNA-Seq protocols using RNA extracted from *Saccharomyces cerevisiae* the dUTP second strand method was identified as the leading protocol [16] and it is most prevalent strand specific RNA-Seq protocol seen in the scientific literature [62-66].

## 6. CONCLUDING REMARKS

Antisense transcription and the expression of antisense transcripts add another layer of complexity to the transcriptome and gene regulation. The modes of regulation by antisense transcripts are diverse and, as a greater amount of research is directed to this area, it is expected that more modes of regulation will be revealed. Strand-specific RNA-Seq holds the key to fully understand the antisense transcriptome. 

When using RNA-Seq to annotate the transcriptome of higher eukaryotic organisms the following might be suggested. Ribosomal RNA depletion should be used for the selection of the RNA fraction to be sequenced, random hexamers for the generation of double-stranded cDNA libraries and strand-specific RNA-Seq for sequencing. By following these guidelines the entirety of the transcriptome can be annotated including the vital non-processed RNAs, such as ncRNAs and the strand of origin information will be retained. These recommendations might help to avoid generation of a limited snapshot of the transcriptome consisting almost entirely of protein coding mRNAs and processed RNAs, which does not fully demonstrate the complexity of the transcriptome.

## Figures and Tables

**Table 1.** Summary of Strand-Specific RNA-Seq Methods.

Method	Advantages	Disadvantages	References
Strand-specific 3'-end RNA-Seq	Technically simple. Follows standard RNA-Seq protocol.	Only polyadenylated mRNA selected. Alignment process may be laborious and difficult.	[54]
Single-stranded adapter ligation	No need for second strand cDNA synthesis. Simplified RNA-Seq protocol. No chemical modification of transcripts.	T4 DNA ligase inefficiently ligates adapters to fragments.	[56]
Flowcell reverse transcription sequencing	No PCR bias. Compatible with paired-end sequencing.	High initial RNA input. Only polyadenylated mRNA selected.	[58, 59]
Bisulfite Treatment	Small modification to standard RNA-Seq protocol.	Sequence alignment process can be difficult.	[16, 19, 61]
dUTP second strand	Small modification to standard RNA-Seq protocol. Rated as the most comprehensive strand-specific sequencing method.	Complex and time-consuming template preparation protocol.	[16, 62-66]
